# Adult Circumcision for Symptomatic Phimosis in Poland: Six-Month Patient-Reported Sexual Function and Psychosocial Outcomes from a Central European Low-Circumcision Setting

**DOI:** 10.3390/jcm15093499

**Published:** 2026-05-02

**Authors:** Michał Falis, Michalina Grudzińska, Weronika Michałowska, Michał Wróbel, Julia Świechowska, Hubert Dyrszka, Michał Bałenkowski, Marcin Matuszewski, Mateusz Czajkowski

**Affiliations:** 1 Department of Urology, Medical University of Gdańsk, Mariana Smoluchowskiego 17 Street, 80-214 Gdańsk, Poland; m.falis@gumed.edu.pl (M.F.); weronika.michalowska@gumed.edu.pl (W.M.); wrobel.michal98@gmail.com (M.W.); julia.swiechowska@gmail.com (J.Ś.); balenkowskimichal@gmail.com (M.B.); marcin.matuszewski@gumed.edu.pl (M.M.); 2 Student Scientific Circle at the Department of Urology, Faculty of Medicine, Medical University of Gdańsk, Marii Skłodowskiej-Curie 3a, 80-210 Gdańsk, Poland; michalinagrudzinska@gumed.edu.pl (M.G.); hubert.dyrszka@gumed.edu.pl (H.D.); 3 Penile Disease Center, University Clinical Center of Gdańsk, Mariana Smoluchowskiego 17 Street, 80-214 Gdańsk, Poland

**Keywords:** male circumcision, phimosis, erectile dysfunction, dyspareunia, sexual function, genital self-image, patient-reported outcomes, depressive symptoms

## Abstract

**Background**: Adult circumcision is an effective surgical treatment for symptomatic phimosis, but its effects on sexual function and psychosocial outcomes remain incompletely characterized. This study aimed to assess patient-reported motivations for circumcision due to phimosis and to evaluate changes in sexual function, genital self-image, and depressive symptoms after surgery. **Methods**: We performed a prospective, single-center observational pre–post cohort study of adult men who underwent circumcision for symptomatic phimosis at a tertiary academic center in Poland between January 2023 and April 2025. Participants completed an author-designed questionnaire and validated patient-reported outcome measures before surgery and at six months after circumcision. The primary endpoint was the change in sexual function measured by the International Index of Erectile Function (IIEF-15). Secondary endpoints included changes in genital self-image assessed using the Male Genital Self-Image Scale (MGSIS-7), depressive symptoms measured with the Beck Depression Inventory (BDI-II), and patient-reported motivations for surgery. Paired comparisons were performed using the Wilcoxon signed-rank test. **Results**: A total of 148 men were included in the study. The most common reasons for surgery were pain/discomfort (58/148, 39.2%) and impaired sexual activity (51/148, 34.5%). The total IIEF-15 score increased from 46.4 ± 20.4 to 57.5 ± 21.0 (*p* < 0.001), with significant improvements across all domains (all *p* < 0.001). The total MGSIS-7 score improved from 19.9 ± 4.5 to 23.1 ± 4.0 (*p* < 0.001). Paired BDI-II data were available for 77 men; the total BDI-II score decreased from 1.2 ± 3.8 to 0.3 ± 1.6 (*p* = 0.004). Most men reported improved sexual life (127/148, 85.8%) and cosmetic satisfaction (124/148, 83.8%), and 119/148 (80.4%) reported no complications. **Conclusions**: Circumcision for symptomatic phimosis was associated with improved sexual function and genital self-image, alongside a statistically significant but clinically negligible decrease in subclinical depressive symptoms.

## 1. Introduction

Phimosis is characterized by the inability to retract the foreskin beyond the glans penis owing to constriction of the preputial ring. It is categorized into physiological phimosis, also known as primary phimosis, which predominantly occurs in the pediatric population, and pathological or secondary phimosis, which is more commonly observed in adults [1,2]. The differentiation between the two subtypes is predicated on the presence of scar tissue in the pathological phimosis. The formation of this scar tissue is facilitated by inadequate genital hygiene, recurrent inflammation of the glans and foreskin, lichen sclerosus, frequent transurethral bladder catheterization, and microtrauma to the foreskin [1,3,4,5].

Among the causes of inability to retract the foreskin, physiological adhesion of the foreskin should also be noted [3]. It is estimated to affect up to 63% of 6–7-year-olds and 3% of 16–17-year-olds without phimosis [6]. Accurate differentiation between these two types of phimosis and foreskin adhesion on physical examination is essential, as it determines the appropriate therapeutic approach [3,4]. In line with current European Association of Urology guidance, non-retractability related to physiological development does not require treatment in asymptomatic cases; forced retraction should be avoided because it may cause scarring and secondary pathological phimosis. Symptomatic phimosis may initially be treated with topical corticosteroids, whereas circumcision is recommended in cases of balanitis xerotica obliterans or phimosis refractory to conservative treatment [3].

Circumcision is one of the oldest known surgical procedures and has been performed for medical, religious, and cultural reasons. Historical sources indicate that the practice has been documented since antiquity, including ancient Egyptian depictions, and it continues to hold religious significance in Judaism and Islam as well as cultural significance in various societies [7,8,9,10]. Over time, the medical rationale for circumcision has evolved substantially. Contemporary medical indications include pathological phimosis, recurrent balanoposthitis refractory to conservative treatment, selected penile premalignant or malignant conditions, and patient-specific functional or hygienic indications [11]. The procedure may be performed under local or general anesthesia depending on age, clinical circumstances, patient preference, and institutional practice. Surgical approaches include conventional excision techniques and device-assisted methods such as Plastibell, Gomco, and PrePex devices [12,13].

The global prevalence of phimosis among adults is estimated to be approximately 3.4% [2]. Despite this relatively low proportion, it remains a significant epidemiological problem that affects numerous aspects of daily life. The inability to retract the foreskin hinders adequate penile hygiene, predisposing patients to balanoposthitis, urinary retention, and urinary tract infections [2]. Symptoms such as pain, foreskin irritation, bleeding, dysuria, and weak urinary stream adversely affect mental health, thereby increasing the risk of anxiety and depression [4,14]. Additionally, discomfort and pain during erection and sexual intercourse significantly impair sexual function [15]. Furthermore, phimosis has been shown to be one of the major risk factors for the development of penile cancer, correlating with more advanced disease at the time of diagnosis and poorer oncological outcomes [16]. In light of these findings, appropriate management of phimosis, including surgical interventions such as MC, is crucial not only for alleviating symptoms but also for enhancing sexual function and overall quality of life.

Nevertheless, despite having symptomatic phimosis, many patients are reluctant to undergo MC because of concerns about a potential loss of sexual pleasure and fear related to the procedure itself. This barrier may be particularly pronounced in countries where circumcision is uncommon and societal familiarity with the procedure is limited. It is estimated that approximately 37–39% of men are circumcised globally, whereas in the general Polish population, this figure is as low as 0.11%, making it one of the lowest rates worldwide [17]. In European literature, the impact of MC on sexual function, mental health, and quality of life remains ambiguous, and data concerning the Polish population are markedly limited.

To address this knowledge gap and provide evidence to support preoperative counselling in low-circumcision settings, we aimed to describe patients’ motivations for surgery and postoperative satisfaction, and to quantify changes in sexual function, genital self-image, and depressive symptoms following circumcision for symptomatic phimosis. Our primary research question was whether circumcision for symptomatic phimosis is associated with a statistically significant improvement in overall sexual function, measured by the total IIEF-15 score, at six months postoperatively. Secondary questions addressed changes in erectile function, genital self-image, depressive symptom scores, postoperative satisfaction, and patient-reported complications.

## 2. Materials and Methods

### 2.1. Patient Selection and Data Collection

This single-center, prospective, observational cohort study was conducted at a tertiary academic urology center in Poland, from January 2023 to April 2025. This study analyzed routinely collected, prospectively administered patient-reported outcome measures. The study protocol was approved by an independent Ethics Committee (decision No. NKBBN/369/2017). This study was conducted in accordance with the Declaration of Helsinki and relevant national regulations. Written informed consent for participation and for the use of clinical and questionnaire data for scientific publication was obtained from all participants.

Eligible participants were adult men (≥18 years) diagnosed with symptomatic (pathological) phimosis and scheduled for circumcision during the study period. Patients aged <18 years or those who did not provide informed consent were excluded. Men who did not undergo circumcision (e.g., those who declined surgery or were not qualified for surgery) were excluded from the cohort.

Data were collected using four survey-based instruments administered at two time points: preoperatively (baseline) and at postoperative follow-up, six months after MC. The instruments comprised an author-designed questionnaire (items 1–19) assessing general characteristics and phimosis-related history, symptoms and sexual difficulties, motivations for surgery, postoperative satisfaction, perceived changes in self-esteem, wound-healing duration, and patient-reported complications (Supplementary Table S1), and three validated questionnaires: IIEF-15 [18], MGSIS-7 [19], and BDI-II [20].

Analyses were performed on paired complete cases for each instrument; therefore, the analytic sample size varied across outcomes. Paired pre–post data were available for 148 participants for the author-designed questionnaire, IIEF-15, and MGSIS-7, and for 77 participants for the BDI-II. Missing BDI-II data occurred because 71 participants did not grant permission to complete this specific questionnaire. Because the reason for missingness could be related to participant characteristics and could not be assumed to be completely at random, BDI-II findings were interpreted cautiously and analyzed as exploratory. No imputation of missing questionnaire data was performed.

The primary outcome was the change in the total IIEF-15 score from baseline to the 6-month follow-up. Secondary outcomes included changes in the IIEF-15 domain scores: erectile function (EF), orgasmic function (OF), sexual desire (SXD), intercourse satisfaction (IS), and overall satisfaction (OS), changes in the total and item-level MGSIS-7 scores, changes in the total and item-level BDI-II scores, and patient-reported satisfaction and complications collected using the author-designed questionnaire.

Circumcision was performed by consultant urologists according to the standard practices of our department under aseptic operating-room conditions. After antiseptic skin preparation and sterile draping, preputial adhesions, when present, were gently released and the phimotic ring together with the redundant foreskin was excised using a conventional open circumcision technique. Hemostasis was secured intraoperatively, and the wound edges were approximated in the standard fashion with absorbable sutures. The specific surgical technique and anesthesia type were selected by the operating surgeon based on patient- and procedure-related factors. Standard postoperative instructions and analgesia were administered. Participants were routinely followed up postoperatively, and follow-up questionnaires were completed six months after surgery.

This study was conducted in accordance with the Strengthening the Reporting of Observational Studies in Epidemiology (STROBE) guidelines to ensure transparency and completeness of reporting [21]. The corresponding STROBE checklist for cohort studies is provided in Supplementary Table S2 [22].

### 2.2. Statistical Methods

All analyses were performed using STATISTICA version 12.5 (StatSoft, Inc., Tulsa, OK, USA). Continuous variables are reported as mean ± standard deviation (SD) and, where appropriate, median with interquartile range (IQR). Categorical variables are presented as counts and percentages. For author-designed questionnaire items allowing multiple responses, each response option was summarized separately; therefore, percentages may not sum to 100%.

The primary outcome was the within-subject change in the total IIEF-15 score from baseline (preoperative) to postoperative follow-up (6 months). Secondary outcomes included changes in the IIEF-15 domain scores (EF, OF, SXD, IS, and OS), changes in the total and item-level MGSIS-7 scores, and changes in the total and item-level BDI-II scores. Patient-reported satisfaction, perioperative experience, and complications derived from the author-designed questionnaire were analyzed descriptively.

Because questionnaire outcomes were based on Likert-type ordinal response options and were not assumed to follow a normal distribution (notably with floor effects for depressive symptom scores), pre- and postoperative comparisons were conducted using the Wilcoxon signed-rank test (paired, two-sided). A *p*-value < 0.050 was considered statistically significant. Due to multiple domain- and item-level comparisons, item-level *p*-values are reported for exploratory purposes; no formal adjustment for multiple testing was applied.

Analyses were conducted using complete paired cases for each instrument. Only participants with evaluable pre- and postoperative scores for a given instrument were included in the corresponding analysis, and no imputation of missing questionnaire items was performed. In cases where the Wilcoxon test could not be computed due to zero variability in paired responses (all paired differences equal to zero), the *p*-value was reported as not determined. No multivariable adjustment was undertaken because the main objective was within-subject pre–post comparison and the study did not include a non-circumcised control group.

## 3. Results

### 3.1. Patient Characteristics

**Figure 1:**
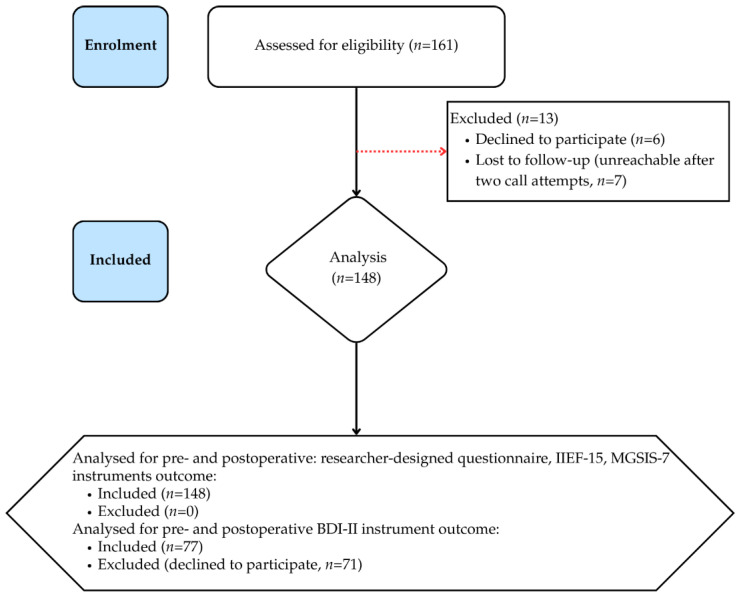
Flow chart of patient selection. BDI-II: Beck Depression Inventory; IIEF-15: International Index of Erectile Function; MGSIS-7: Male Genital Self-Image Scale.

A total of 161 men diagnosed with symptomatic phimosis who underwent MC in our department were assessed for eligibility. Of these, 7 were lost to follow-up after two unsuccessful telephone contact attempts and 6 were excluded because they did not provide informed consent. The remaining 148 participants were included in the paired analyses for the author-designed questionnaire, IIEF-15, and MGSIS-7; paired BDI-II data were available for 77 participants. A flow chart of the study cohort selection is provided in Figure 1. Among the final cohort of 148 participants, the mean age at the time of surgery was 38.3 ± 19.0 years (median, 31.6 years). Respondents most commonly lived in large cities (>500,000 residents, 90/148, 60.8%; 200,000–500,000 residents, 27/148, 18.2%; 100,000–200,000 residents, 9/148, 6.1%), 12/148 (8.1%) lived in cities up to 50,000 residents, and 10/148 (6.8%) lived in the countryside. Nearly half of the patients had higher education (74/148; 50.0%), 63/148 (42.6%) had secondary education, 6/148 (4.1%) had primary education, and 5/148 (3.4%) of study participants had vocational education. Among the men, 70/148 (47.3%) were married, 53/148 (35.8%) were single, and 25/148 (16.9%) were in a partnership. Of the patients, 125/148 (84.5%) reported having a current sexual partner. The demographic characteristics of the cohort are shown in Table 1.

### 3.2. Patient-Reported Motivations for Circumcision

Among the patients, 29.1% (*n* = 43) reported multiple reasons for undergoing the procedure. The predominant motivations for undergoing MC were pain or discomfort (58/148, 39.2%), impaired sexual activity (51/148, 34.5%), and genital inflammation (15/148, 10.1%). Four participants (2.7%) underwent the procedure based on their physician’s recommendation. Overall, MC was performed for medical or sexual health reasons in 111 cases (75%).

### 3.3. Preoperative Symptoms and Sexual Difficulties

Prior to undergoing surgical intervention, a substantial majority of participants (109/148, 73.6%) reported experiencing discomfort or pain, characterized by sensations such as burning or an unpleasant feeling during sexual intercourse. Conversely, 39/148 (26.4%) participants did not report this issue (Table 2). Participants were queried regarding whether phimosis impeded sexual intercourse. Of the 148 men surveyed, 112 (75.7%) responded affirmatively, indicating a generally adverse effect on sexual activity. Furthermore, the initiation of sexual activity was reported to be obstructed by phimosis in 96 out of 148 men (64.9%) (Table 2).

Prior to surgery, a considerable number of patients experienced reduced sexual activity due to the disease. Intercourse occurred at least once a day was reported in 4/148 (2.7%) participants, at least once per week in 58/148 (39.2%) men and at least once per month in 46/148 (31.1%) men. A total of 13 of 148 participants (8.8%) engaged in sexual activity no more than once annually, whereas 27/148 (18.2%) reported complete abstinence.

Among the 148 respondents, the onset of phimosis symptoms was reported more than a year before surgery by 76 (51.4%) participants. In 65/148 (43.9%) men, symptoms appeared within the year preceding the procedure. Specifically, the majority of these patients fell within the 6–12 months range (34/148, 23.0%). This was followed by 27/148 participants (18.2%) who reported symptoms in the 2–6 months interval, and 4/148 (2.7%) in the 1–4 weeks range. No patients reported symptom onset of one week or less. Moreover, 3/148 (2.0%) individuals experienced no symptoms before the MC and 4/148 (2.7%) had not had sexual intercourse.

### 3.4. Subjective Outcomes of Surgery

Following therapeutic intervention, 127/148 (85.8%) participants reported an improvement in their sexual life. Conversely, 17/148 (11.5%) indicated no change, while 4/148 (2.7%) reported worsening. Regarding self-esteem, 64/148 (43.2%) men reported that circumcision affected their self-esteem (Yes/Rather yes). Among those, 59/64 (92.2%) reported that the effect was positive. A cosmetically satisfactory outcome was reported by 124/148 (83.8%) patients. Furthermore, 141/148 (95.3%) participants declared that they would recommend circumcision to individuals experiencing similar problems (Table 3).

The postoperative healing period typically spanned from two to four weeks. Notably, wound healing within a single week was reported by 6/148 participants (4.1%), while 58/148 (39.2%) required two weeks. The predominant healing duration was one month, including 69/148 (46.6%) of respondents. Beyond this peak, 11/148 (7.4%) indicated two months and 4/148 (2.7%) reported a period exceeding two months.

No complications were reported in 119 (80.4%) of the 148 patients. Complications were reported in 29/148 (19.6%) patients. Since complication data were patient-reported rather than extracted from medical records, formal Clavien–Dindo grading was not always possible; however, most complications appeared consistent with Grade I (transient, requiring no pharmacological or surgical intervention) [23]. The most frequently observed complications included temporary hemorrhage (18/29, 62.1%) and painful erections (6/29, 20.7%) which, along with edema (1/29, 3.4%), were transient and resolved within several days to two weeks. Additional adverse events included difficulties with penetration (2/29, 6.9%), decreased libido (1/29, 3.4%) and keloid scar (1/29, 3.4%), and the latter could correspond to a higher Clavien–Dindo grade only if further surgical or procedural intervention was required; otherwise, precise grading could not be determined from the available patient-reported data [23].

### 3.5. IIEF-15: Erectile Function and Sexual Domains

Using the IIEF-15 questionnaire [18], five domains of sexual functioning were assessed: EF, OF, SXD, IS, and OS. Erectile dysfunction (ED) severity was classified using established cutoffs for the EF domain (range, 6–30): severe (6–10), moderate (11–16), mild-to-moderate (17–21), mild (22–25), and no ED (26–30) [24].

Prior to medical consultation, the distribution of all 148 patients across the specified categories was as follows: 27 (18.2%) were classified as severe; 10 (6.8%) as moderate; 16 (10.8%) as mild-to-moderate; 29 (19.6%) as mild; and 66 (44.6%) exhibited no ED. At six months after circumcision, the mean EF domain score increased by 3.7 points. Although this finding should be interpreted in the context of the study’s observational design and potential selection bias, the observed improvement exceeded the cohort’s weighted Minimal Clinically Important Difference (MCID) threshold of 2.2 points based on baseline ED severity [25]. This suggests that the change in EF domain score was not only statistically significant (*p* < 0.001), but also clinically meaningful at the cohort level. The post-treatment classification of patients by ED severity was as follows: 21 (14.2%) were categorized as severe; 2 (1.4%) as moderate; 4 (2.7%) as mild-to-moderate; 23 (15.5%) as mild; and 98 (66.2%) exhibited no ED. Consistently, the mean total IIEF-15 score increased from 46.4 ± 20.4 (median, 53; IQR, 28–63) preoperatively to 57.5 ± 21.0 (median, 68; IQR, 55–70) postoperatively (*p* < 0.001; Table 4).

Before MC, 69 patients reported no problems with OF (46.6% of the respondents who achieved the maximum score on these items). After MC, 106 men achieved the maximum score in this category (71.6%).

After analyzing the results regarding SXD, a significant increase was observed. Before the procedure, only 10 (6.8%) patients achieved the maximum score for these items. Following this procedure, as many as 20 (13.5%) patients achieved this outcome. All IIEF-15 domains (EF, OF, SXD, IS, and OS) showed clear postoperative improvement in paired analyses (Table 4).

### 3.6. Genital Self-Image (MGSIS-7)

Among the 148 men who completed the MGSIS-7 at both time points, genital self-image significantly improved after surgery. The mean total MGSIS-7 score increased from 19.9 ± 4.5 (median 21; IQR 17–22) preoperatively to 23.1 ± 4.0 (median 21; IQR 21–28) postoperatively (*p* < 0.001; Table 5).

### 3.7. Depressive Symptoms (BDI-II)

Paired BDI-II data were available for 77/148 participants (52.0%). At baseline, most participants reported very low depressive symptom scores, consistent with a pronounced floor effect (mean 1.2 ± 3.8, median 0). Postoperatively, the mean total BDI-II score decreased from 1.2 ± 3.8 (median 0) to 0.3 ± 1.6 (median 0) (*p* = 0.004). Given the very low baseline symptom burden, the high proportion of missing paired BDI-II data, and median scores of zero at both time points, this statistically significant change should be considered exploratory and not clinically meaningful. The observed significance likely reflects minor score fluctuations in a small subset of participants rather than a clinically relevant antidepressant effect of surgery. Detailed item-level BDI-II results are provided in Supplementary Table S3.

## 4. Discussion

In this single-center, prospective observational pre–post cohort study, we investigated adult men undergoing circumcision for symptomatic phimosis in a low-circumcision setting in Poland. Our objectives were to characterize patient motivations for surgery, describe perioperative experience (including healing and complications), and evaluate changes in sexual function and selected psychosocial outcomes from the preoperative baseline to postoperative follow-up using a researcher-designed questionnaire and validated instruments (IIEF-15, MGSIS-7, and BDI-II). Overall, circumcision performed for phimosis was associated with improved patient-reported sexual function (higher IIEF-15 total and domain scores), improved genital self-image, and a modest reduction in depressive symptom scores, accompanied by high satisfaction and a low incidence of clinically significant postoperative complications.

According to the Declaration of Sexual Rights published by the World Association for Sexual Health, human sexuality constitutes a fundamental aspect of life throughout the entire lifespan and serves as a source of pleasure and well-being that contributes to overall fulfilment and satisfaction with life [26]. Previous studies have demonstrated an association between sexual satisfaction and mental health, with lower levels of sexual satisfaction linked to an increased risk of depressive and anxiety symptoms [27]. Available evidence further suggests that a substantial proportion of men report dissatisfaction with the quality of their sexual life, with published estimates ranging between 15% and 41% [28]. Among potentially modifiable contributors to sexual dissatisfaction, phimosis is clinically relevant because it is frequently associated with pain during erection, discomfort during intercourse, and avoidance of sexual activity [15]. Consequently, effective treatment of phimosis, most commonly by MC, may plausibly alleviate these symptoms and translate into improved patient-reported sexual outcomes.

Importantly, the impact of adult MC on sexual function remains heterogeneous across studies, reflecting differences in indications for surgery, baseline sexual function, age, cultural context, surgical technique, and outcome assessment. In a Polish cohort study by Czajkowski et al. (*n* = 67; mean age 45.1 ± 20 years), MC was associated with improved sexual function as reflected by an increase in IIEF-5 scores, and the authors also reported improved genital self-image assessed with the same MGSIS-7 tool used in our study [29]. In 2017, Nordström et al. conducted a large, controlled study in an African population (1509 newly circumcised men and 1524 age-matched uncircumcised controls) and found that MC either improved sexual function and pleasure or resulted in no measurable change [30]. In line with the notion that MC is not inherently detrimental to erectile function, Hoschke et al. analyzed 167 cases using the validated IIEF questionnaire and demonstrated that MC was not associated with an increased prevalence of erectile dysfunction [31]. A recent meta-analysis by Karaahmet et al. similarly reported increased sexual satisfaction and a modest but statistically significant improvement in erectile function, while emphasizing substantial heterogeneity among included studies [32]. Conversely, Kim et al. reported less favorable outcomes in a cohort of 373 sexually active men (255 circumcised and 118 uncircumcised) circumcised after the age of 20 years, in which only 6% reported improved sexual quality of life and 20% reported deterioration; the authors also observed a high proportion of men reporting reduced pleasure during masturbation [33]. Collectively, these data underscore that adult MC cannot be discussed as a uniform exposure and that outcomes may depend strongly on indication and context.

To place these findings in a broader psychosexual context, circumcision may be considered alongside other penile surgical interventions, although outcomes differ substantially according to indication, invasiveness, baseline sexual function, and patient expectations. More extensive procedures, such as partial penectomy for penile cancer, have been associated with significant sexual and psychosexual burden, including concerns about penile appearance and perceived loss of masculinity [34]. Patients with Peyronie’s disease may also experience emotional distress, depressive symptoms, body-image concerns, relationship difficulties, and avoidance of intimacy, indicating that anatomical penile conditions can have effects extending beyond physical symptoms [35,36]. Similarly, after surgical repair of penile fracture, some patients report persistent fear of recurrence and behavioral modifications despite preserved vascular function [37]. In contrast, less destructive or function-restoring procedures may be associated with maintained or improved sexual outcomes. Systematic reviews of circumcision suggest that the procedure is unlikely to adversely affect male sexual function in most studied populations and may improve selected domains in appropriately indicated patients [38,39]. Long-term data after childhood hypospadias repair also indicate generally favorable sexual and psychosexual outcomes in many men, although results vary by severity and surgical history [40,41]. These findings support the interpretation that penile surgery should not be treated as a uniform exposure; psychosexual outcomes depend strongly on the underlying condition, the extent of surgery, and the patient’s preoperative symptom burden. Against this backdrop, our study adds evidence from a low-circumcision European setting and, to the best of our knowledge, represents the largest cohort of this kind in a Polish population. In a cohort restricted to men undergoing MC for symptomatic phimosis, we observed significant postoperative improvements in sexual function and satisfaction, reflected by higher IIEF-15 scores across all domains. This pattern is clinically plausible in phimosis, where alleviation of pain, mechanical restriction, and anxiety related to intercourse may preferentially improve intercourse satisfaction and overall satisfaction and secondarily contribute to improvements in erectile and orgasmic domains. At the same time, because a relevant proportion of participants reported sexual abstinence at baseline and/or follow-up, IIEF-15 findings particularly within the IS and OS domains should be interpreted in the context of incomplete sexual activity, which may influence domain-specific scoring and the comparability of responses over time.

Beyond sexual functioning, body image and genital self-perception are important determinants of sexual confidence and satisfaction. Previous studies have highlighted associations between negative body attitudes, self-consciousness during intimacy, and sexual dissatisfaction in men [28]. In this context, the observed postoperative improvement in MGSIS-7 scores in our cohort suggests enhanced genital self-image after resolution of phimosis, which may facilitate greater comfort during sexual activity and contribute to improved sexual satisfaction. Although cosmetic satisfaction after MC is generally high, published results vary across cohorts and indications. Accordingly, Cortés et al. reported cosmetic satisfaction in 95.5% of a 22-patient cohort [42], and Czajkowski et al. similarly documented significant postoperative improvement in genital self-image [29]. Our findings are concordant with these reports, supporting the relevance of genital self-image as a meaningful patient-reported outcome after phimosis surgery. Notably, in our cohort, high cosmetic satisfaction and willingness to recommend surgery were observed in the context of a relatively low complication rate and a generally short self-reported healing time, suggesting that an uncomplicated postoperative course may be an important contributor to favorable patient experience and perceived benefit.

With respect to psychological well-being, the association between sexual functioning and mental health is well established, including links between reduced sexual satisfaction and increased depressive or anxiety symptoms [27]. In our cohort, BDI-II scores decreased after surgery; however, the very low baseline prevalence of depressive symptoms suggests a floor effect and limits conclusions regarding clinical relevance. Nevertheless, prior evidence indicates that MC may influence psychological domains in selected populations. Aydoğmuş et al. reported a reduction in anxiety levels following MC, with circumcised men exhibiting lower anxiety levels compared to controls [43]. This finding supports the broader hypothesis that addressing a distressing genital condition may offer psychological benefits to at least some patients. Mechanistically, our results are compatible with a model in which symptom relief and improved genital self-image contribute to improved sexual satisfaction and related aspects of well-being, and the observed relationship between cosmetic satisfaction and sexual satisfaction may reflect a mediating pathway whereby changes in perceived appearance influence body image, confidence, sexual functioning, and overall quality of life [44]. While formal mediation analyses were beyond the scope of the present study, future research could explicitly test these pathways and identify which patients benefit most.

Among the strengths of this study are its prospective design and the relatively large cohort (*n* = 148), which, to the best of our knowledge, represents the largest analysis of this topic in the Polish population and one of the larger European cohorts, thereby addressing an important gap in the literature. In addition, the use of validated, standardized patient-reported outcome measures alongside an author-designed questionnaire (IIEF-15, MGSIS-7, and BDI-II) and their administration both preoperatively and postoperatively enabled a comprehensive within-subject comparison, strengthening the internal consistency of the observed changes over time. Key limitations include the single-center, observational pre–post design without a control group and the reliance on self-reported questionnaire data, which are inherently subjective and vulnerable to reporting and response biases. While the observed improvements are statistically robust, the absence of a control group prevents a definitive causal claim; however, the six-month follow-up reduces the likelihood that the findings merely reflect immediate postoperative fluctuations, and it does not eliminate regression to the mean or expectancy effects, which remain important limitations of the uncontrolled pre–post design. Furthermore, the findings may be influenced by selection bias, as the enrollment of a treatment-seeking population might overrepresent individuals predisposed to reporting favorable outcomes. The single-center setting and distinct demographic profile of the cohort also limit the broader external validity of these results. Data completeness was also limited by partial non-response and missing paired follow-up data (particularly for BDI-II), which may reduce generalizability to non-responders despite statistically significant findings. In addition, sexual inactivity in a subset of participants represents an important interpretive context for patient-reported sexual outcomes, especially those domains more directly dependent on intercourse. Finally, the absence of objective measures of erectile function and biological correlates (e.g., hormonal assessments) limits causal inference; therefore, future research should prioritize multicenter designs with an appropriate control group, objective functional assessments, hormonal analyses, and partner-reported outcomes to further strengthen the evidence base.

## 5. Conclusions

In adult men undergoing surgical treatment for symptomatic phimosis, circumcision was associated with improvements in sexual function, satisfaction, and genital self-image, as well as a statistically significant but clinically negligible decrease in subclinical depressive symptom scores. The most common motivations for seeking treatment were pain and sexual difficulties, while most participants reported high postoperative satisfaction and uncomplicated healing. Because this was a single-center observational pre–post study without a control group, these findings should be interpreted as associations rather than definitive causal effects and should be considered within the context of a motivated treatment-seeking population in a low-circumcision European setting. Further controlled studies with objective outcome measures are needed to better establish causality.

## Figures and Tables

**Table 1:** Demographic characteristics of the study cohort.

Variable	Category	*n*	%
Age	18–24	53	35.8%
	25–34	32	21.6%
	35–44	19	12.8%
	45–54	11	7.4%
	≥55	33	22.3%
Place of residence (city size)	Rural area	10	6.8%
	Town/city up to 50,000	12	8.1%
	Town/city 100,000–200,000	9	6.1%
	Town/city 200,000–500,000	27	18.2%
	City over 500,000	90	60.8%
Level of education	Vocational	5	3.4%
	Primary	6	4.1%
	Secondary	63	42.6%
	Higher	74	50.0%
Marital status	Single	53	35.8%
	Partnership	25	16.9%
	Married	70	47.3%
Steady sexual partner	Yes	125	84.5%
	No	23	15.5%

**Table 2:** The results from the author-designed pre-circumcision questionnaire.

PREOPERATIVE						
Question	Yes	Rather Yes	No Change	Rather No	No	Other Responses
1. Before circumcision, did you experience any discomfort or pain, such as burning or unpleasant sensation during sexual intercourse?	82 (55.4%)	27 (18.2%)	2 (1.4%)	13 (8.8%)	20 (13.5%)	4 (2.7%)–no sexual activity
2. Did the presence of phimosis negatively affect your sexual life?	84 (56.8%)	28 (18.9%)	1 (0.7%)	14 (9.5%)	17 (11.5%)	4 (2.7%)–no sexual activity
3. Did you have difficulty initiating sexual intercourse due to phimosis?	66 (44.6%)	30 (20.3%)	11 (7.4%)	24 (16.2%)	13 (8.8%)	4 (2.7%)–no sexual activity

**Table 3:** The results from the author-designed post-circumcision questionnaire.

POSTOPERATIVE						
Question	Yes	Rather Yes	No Change	Rather No	No	Other Responses
1. Did circumcision positively affect your sexual life?	74 (50.0%)	53 (35.8%)	17 (11.5%)	2 (1.4%)	2 (1.4%)	-
2. Did circumcision affect your self-esteem?	47 (31.8%)	17 (11.5%)	76 (51.4%)	4 (2.7%)	4 (2.7%)	-
3. If you answered that the procedure had affected your self-esteem (answer Yes/Rather yes), was this effect positive?	59 (39.9%)	-	-	-	5 (3.4%)	Not applicable 84 (56.8%)
4. Would you recommend circumcision to individuals experiencing problems similar to yours?	134 (90.5%)	7 (4.7%)	4 (2.7%)	1 (0.7%)	2 (1.4%)	-
5. Are you content with the cosmetically satisfactory outcome?	116 (78.4%)	8 (5.4%)	11 (7.4%)	7 (4.7%)	6 (4.1%)	-

**Table 4:** IIEF-15—preoperative and postoperative results.

Domain	Preoperative	Postoperative	*p*-Value *
EF (6–30)	18.2 ± 9.6 median 22 (IQR 10–26)	21.9 ± 9.2 median 25 (IQR 21–29)	<0.001
OF (2–10)	6.4 ± 4.0 median 8 (IQR 2–10)	8.0 ± 3.6 median 10 (IQR 8–10)	<0.001
SXD (2–10)	7.0 ± 2.1 Median 7 (IQR 6–9)	7.8 ± 2.0 median 8 (IQR 7–9)	<0.001
IS (3–15)	8.4 ± 5.5 median 9 (IQR 3–14)	11.5 ± 5.4 median 14.5 (IQR 12–15)	<0.001
OS (2–10)	6.4 ± 2.2 median 7 (IQR 5–8)	8.3 ± 2.0 median 8 (IQR 8–10)	<0.001
Total	46.4 ± 20.4 median 53 (IQR 28–63)	57.5 ± 21.0 median 68 (IQR 55–70)	<0.001

* IQR, Interquartile Range; EF, erectile function; OF, orgasmic function; SXD, sexual desire; IS, intercourse satisfaction; OS, overall satisfaction.

**Table 5:** MGSIS-7—preoperative and postoperative results.

	Preoperative (mean ± SD)	Postoperative (mean ± SD)	*p*-Value *
1. I feel positively about my genitals.	2.8 ± 0.9	3.3 ± 0.7	<0.001
2. I am satisfied with the appearance of my genitals.	2.8 ± 0.9	3.3 ± 0.6	<0.001
3. I would feel comfortable letting a sexual partner look at my genitals.	2.8 ± 0.9	3.3 ± 0.7	<0.001
4. I am satisfied with the size of my genitals.	3.2 ± 0.7	3.3 ± 0.7	0.020
5. I think my genitals work the way they are supposed to work.	2.6 ± 0.8	3.4 ± 0.7	<0.001
6. I feel comfortable letting a health care provider examine my genitals.	2.9 ± 0.8	3.2 ± 0.7	<0.001
7. I am not embarrassed about my genitals.	2.8 ± 0.9	3.4 ± 0.6	<0.001
Total	19.9 ± 4.5	23.1 ± 4.0	<0.001

* IQR, Interquartile Range.

## Data Availability

The original contributions presented in this study are included in the article/Supplementary Material. Further inquiries can be directed to the corresponding author.

## Supplementary Materials

The following supporting information can be downloaded at: https://www.mdpi.com/article/10.3390/jcm15093499/s1, Table S1: The Author-Designed General, Pre- and Post-Circumcision Questionnaire; Table S2: STROBE Statement—Checklist of items that should be included in reports of cohort studies. STROBE Statement—checklist of items that should be included in reports of observational studies; Table S3: BDI-II–preoperative and postoperative results.

## Abbreviations

The following abbreviations are used in this manuscript:

BDI-II	Beck Depression Inventory
EAU	European Association of Urology
ED	Erectile dysfunction
EF	Erectile function
GDPR	General Data Protection Regulation
IIEF-15	International Index of Erectile Function
IS	Intercourse satisfaction
IQR	Interquartile range
MC	Male circumcision
MGSIS-7	Male Genital Self-Image Scale
OF	Orgasmic function
OS	Overall satisfaction
SD	Standard deviation
STROBE	Strengthening the Reporting of Observational Studies in Epidemiology
SXD	Sexual desire
