# Supplementary material for: Adult Circumcision for Symptomatic Phimosis in Poland: Six-Month Patient-Reported Sexual Function and Psychosocial Outcomes from a Central European Low-Circumcision Setting
Source: J Clin Med. 2026 May 2;15(9):3499. doi: 10.3390/jcm15093499 (PMC13163725; doi:10.3390/jcm15093499)
Supplement: Supplementary file 1 [file jcm-15-03499-s001.zip › Table S1-edited.pdf]

Supplementary Table S1. The Author-Designed General, Pre- and Post-Circumcision Questionnaire

GENERAL QUESTIONS AND ANSWERS	
How big is your place of residence?	'Rural area'<br>'Town/city under 50,000 inhabitants'<br>'Town/city with 100,000–200,000 inhabitants'<br>'Town/city with 200,000–500,000 inhabitants'<br>'City with more than 500,000 inhabitants'
What is your highest level of education?	'Primary education'<br>'Secondary education'<br>'Higher education'
**PREOPERATIVE QUESTIONS AND ANSWERS**	
What was your marital status?	'Married'<br>'Partnership'<br>'Single'
Did you have a sexual partner at that time?	'Yes'<br>'No'
How old were you when you underwent circumcision?	'18-24'<br>'25-34'<br>'35-44'<br>'45-54'<br>'≥ 55'

Why did you undergo circumcision?	‘Pain/discomfort’<br>‘Impaired sexual activity’<br>‘Difficulty urinating’<br>‘Genital inflammation’<br>‘Medical indication’<br>‘Other, please specify’
Before circumcision, did you experience any discomfort or pain, such as burning or unpleasant sensation during sexual intercourse?	‘Yes’<br>‘Rather yes’<br>‘No change’<br>‘Rather no’<br>‘No’
Did the presence of phimosis negatively affect your sexual life?	‘Yes’<br>‘Rather yes’<br>‘No change’<br>‘Rather no’<br>‘No’
Did you have difficulty initiating sexual intercourse due to phimosis?	‘Yes’<br>‘Rather yes’<br>‘No change’<br>‘Rather no’<br>‘No’
Before circumcision, how often did you have sexual intercourse?	‘Once a day or more’<br>‘Once a week or more’<br>‘Once a month or more’<br>‘Once a year or more’<br>‘None’

When did the first symptoms of phimosis appear before medical treatment?	'One week or less'<br>'One month or less'<br>'Six months or less'<br>'One year or less'<br>'More than a year'<br>'No symptoms'
**POSTOPERATIVE QUESTIONS AND ANSWERS**	
Did circumcision positively affect your sexual life?	'Yes'<br>'Rather yes'<br>'No change'<br>'Rather no'<br>'No'
Did circumcision affect your self-confidence?	'Yes'<br>'Rather yes'<br>'No change'<br>'Rather no'<br>'No'
If you answered that the procedure has affected your self-confidence, was this effect positive?	'Yes'<br>'No'
Who encouraged you to undergo the procedure?	'Myself'<br>'Partner'<br>'Family member'<br>'Doctor'<br>'Other, please specify'

Would you recommend circumcision to individuals experiencing problems similar to yours?	'Yes'<br>'Rather yes'<br>'No change'<br>'Rather no'<br>'No'
Did you experience any complications after circumcision? If so, what were they and how long did they last?	'No'<br>'Yes, please specify'
How long did the wound after circumcision take to heal?	'A week'<br>'Two weeks'<br>'A month'<br>'Two months'<br>'More than two months'
Are you content with the cosmetically satisfactory outcome?	'Yes'<br>'Rather yes'<br>'No change'<br>'Rather no'<br>'No'
