# Supplementary material for: Adult Circumcision for Symptomatic Phimosis in Poland: Six-Month Patient-Reported Sexual Function and Psychosocial Outcomes from a Central European Low-Circumcision Setting
Source: J Clin Med. 2026 May 2;15(9):3499. doi: 10.3390/jcm15093499 (PMC13163725; doi:10.3390/jcm15093499)
Supplement: Supplementary file 1 [file jcm-15-03499-s001.zip › Table S2-edited.pdf]

Supplementary Table S2. STROBE Statement—Checklist of items that should be included in reports of ***cohort studies.*** STROBE Statement—checklist of items that should be included in reports of observational studies

	**Item No.**	**Recommendation**	**Page No.**	**Relevant text from manuscript**
**Title and abstract**	1	(*a*) Indicate the study's design with a commonly used term in the title or the abstract	**1**	"We performed a prospective, single-center observational pre–post cohort study"
		(*b*) Provide in the abstract an informative and balanced summary of what was done and what was found	**1**	"Participants completed an author-designed questionnaire and validated patient-reported outcome measures before surgery and at six months after circumcision."<br><br>"Circumcision for symptomatic phimosis was associated with improved sexual function and genital self-image, alongside a statistically significant but clinically negligible decrease in subclinical depressive symptoms."
**Introduction**				
Background/rationale	2	Explain the scientific background and rationale for the investigation being reported	**3**	"In European literature, the impact of MC on sexual function, mental health, and quality of life remains ambiguous, and data concerning the Polish population are markedly limited."

				“To address this knowledge gap and provide evidence that may support preoperative counselling in low-circumcision settings”
Objectives	3	State specific objectives, including any prespecified hypotheses	**3**	“we aimed to describe patients' motivations for surgery and postoperative satisfaction, and to quantify changes in sexual function, genital self-image, and depressive symptoms following circumcision for symptomatic phimosis. Our primary research question was whether circumcision for symptomatic phimosis is associated with a statistically significant improvement in overall sexual function, measured by the total IIEF-15 score, at six months postoperatively. Secondary questions addressed changes in erectile function, genital self-image, depressive symptom scores, postoperative satisfaction, and patient-reported complications”
**Methods**				
Study design	4	Present key elements of study design early in the paper	**3**	“This single-center, prospective, observational cohort study […]

				analyzed routinely collected, prospectively administered patient-reported outcome measures.”
Setting	5	Describe the setting, locations, and relevant dates, including periods of recruitment, exposure, follow-up, and data collection	**3**	“This single-center, prospective, observational cohort study was conducted at a tertiary academic urology center in Poland, from January 2023 to April 2025. This study analyzed routinely collected, prospectively administered patient-reported outcome measures. The study protocol was approved by an independent Ethics Committee (decision No. NKBBN/369/2017).”<br><br>“Data were collected using four survey-based instruments administered at two time points: preoperatively (baseline) and at postoperative follow-up, six months after MC.”
Participants	6	(*a*) *Cohort study*—Give the eligibility criteria, and the sources and methods of selection of participants. Describe methods of follow-up<br>*Case–control study*—Give the eligibility criteria, and the sources and methods of case ascertainment and control selection. Give the rationale for the choice of cases and controls<br>*Cross-sectional study*—Give the eligibility criteria, and the sources and methods of selection of participants	**3-4**	“Eligible participants were adult men (≥18 years) diagnosed with symptomatic (pathological) phimosis and scheduled for circumcision during the study period. Patients aged < 18 years or those who did not provide informed consent were

				excluded. Men who did not undergo circumcision (e.g., those who declined surgery or were not qualified for surgery) were excluded from the cohort."<br><br>"Participants were routinely followed up postoperatively, and follow-up questionnaires were completed six months after surgery."
		(*b*) *Cohort study*—For matched studies, give matching criteria and number of exposed and unexposed<br>*Case–control study*—For matched studies, give matching criteria and the number of controls per case	**4**	"the study did not include a non-circumcised control group."
Variables	7	Clearly define all outcomes, exposures, predictors, potential confounders, and effect modifiers. Give diagnostic criteria, if applicable	**3-4**	"The primary outcome was the change in the total IIEF-15 score from baseline to the 6-month follow-up. Secondary outcomes included changes in the IIEF-15 domain scores: erectile function (EF), orgasmic function (OF), sexual desire (SXD), intercourse satisfaction (IS), and overall satisfaction (OS), changes in the total and item-level MGSIS-7 scores, changes in the total and item-level BDI-II scores, and patient-reported satisfaction and complications collected using

				the author-designed questionnaire.”<br><br>“The instruments comprised an author-designed questionnaire (items 1–19) assessing general characteristics and phimosis-related history, symptoms and sexual difficulties, motivations for surgery, postoperative satisfaction, perceived changes in self-esteem, wound-healing duration, and patient-reported complications”
Data sources/ measurement	8*	For each variable of interest, give sources of data and details of methods of assessment (measurement). Describe comparability of assessment methods if there is more than one group	**3**	“Data were collected using four survey-based instruments administered at two time points: preoperatively (baseline) and at postoperative follow-up, six months after MC. The instruments comprised an author-designed questionnaire (items 1–19) assessing general characteristics and phimosis-related history, symptoms and sexual difficulties, motivations for surgery, postoperative satisfaction, perceived changes in self-esteem, wound-healing duration, and patient-reported complications (Supplementary Table S1), and three validated

				questionnaires: IIEF-15 [18], MGSIS-7 [19], and BDI-II [20].”
Bias	9	Describe any efforts to address potential sources of bias	**3-4**	“Analyses were performed on paired complete cases for each instrument; therefore, the analytic sample size varied across outcomes.”<br><br>“Circumcision was performed by consultant urologists according to the standard practices of our department under aseptic operating-room conditions.”
Study size	10	Explain how the study size was arrived at	**3-4**	“study was conducted at a tertiary academic urology center in Poland, from January 2023 to April 2025.”<br><br>“Analyses were performed on paired complete cases for each instrument; therefore, the analytic sample size varied across outcomes. Paired pre–post data were available for 148 participants for the author-designed questionnaire, IIEF-15, and MGSIS-7, and for 77 participants for the BDI-II. Missing BDI-II data occurred because 71 participants did not

grant permission to complete this specific questionnaire. Because the reason for missingness could be related to participant characteristics and could not be assumed to be completely at random, BDI-II findings were interpreted cautiously and analyzed as exploratory. No imputation of missing questionnaire data was performed.”

Continued on next page

Quantitative variables	11	Explain how quantitative variables were handled in the analyses. If applicable, describe which groupings were chosen and why	**4**	"Continuous variables are reported as mean ± standard deviation (SD) and, where appropriate, median with interquartile range (IQR). Categorical variables are presented as counts and percentages. For author-designed questionnaire items allowing multiple responses, each response option was summarized separately; therefore, percentages may not sum to 100%."
Statistical methods	12	(*a*) Describe all statistical methods, including those used to control for confounding	**4**	"All analyses were performed using STATISTICA version 12.5 (StatSoft, Inc., Tulsa, OK, USA)."<br><br>"Because questionnaire outcomes were based on Likert-type ordinal response options and were not assumed to follow a normal distribution (notably with floor effects for depressive symptom scores), pre- and postoperative comparisons were conducted using the Wilcoxon signed-rank test (paired, two-sided). A p-value < 0.050 was considered statistically significant. Due to multiple domain- and item-level comparisons, item-level p-values are reported for exploratory purposes; no formal adjustment for multiple testing was applied."

	(*b*) Describe any methods used to examine subgroups and interactions	**4**	"pre- and postoperative comparisons were conducted using the Wilcoxon signed-rank test (paired, two-sided). A p-value < 0.050 was considered statistically significant. Due to multiple domain- and item-level comparisons, item-level p-values are reported for exploratory purposes; no formal adjustment for multiple testing was applied."
	(*c*) Explain how missing data were addressed	**4**	"Analyses were conducted using complete paired cases for each instrument. Only participants with evaluable pre- and postoperative scores for a given instrument were included in the corresponding analysis, and no imputation of missing questionnaire items was performed. In cases where the Wilcoxon test could not be computed due to zero variability in paired responses (all paired differences equal to zero), the p-value was reported as not determined. No multivariable adjustment was undertaken because the main objective was within subject pre–post comparison and the study did not include a non-circumcised control group."
	(*d*) *Cohort study*—If applicable, explain how loss to follow-up was addressed	**-**	n/a

		Case–control study—If applicable, explain how matching of cases and controls was addressed<br>*Cross-sectional study*—If applicable, describe analytical methods taking account of sampling strategy		
		(*e*) Describe any sensitivity analyses	**4**	"no imputation of missing questionnaire items was performed."
**Results**				
Participants	13*	(a) Report numbers of individuals at each stage of study—e.g., numbers potentially eligible, examined for eligibility, confirmed eligible, included in the study, completing follow-up, and analysed	**5**	"A total of 161 men diagnosed with symptomatic phimosis who underwent MC in our department were assessed for eligibility. Of these, 7 were lost to follow-up after two unsuccessful telephone contact attempts and 6 were excluded because they did not provide informed consent. The remaining 148 participants were included in the paired analyses for the author-designed questionnaire, IIEF-15, and MGSIS-7; paired BDI-II data were available for 77 participants. A flow chart of the study cohort selection is provided in Figure 1."
		(b) Give reasons for non-participation at each stage	**5**	"7 were lost to follow-up after two unsuccessful telephone contact attempts and 6 were excluded because they did not provide informed consent."
		(c) Consider use of a flow diagram	**5**	"A flow chart of the study cohort selection is provided in Figure 1."

Descriptive data	14*	(a) Give characteristics of study participants (e.g., demographic, clinical, social) and information on exposures and potential confounders	**5**	"Among the final cohort of 148 participants, the mean age at the time of surgery was 38.3 ± 19.0 years (median, 31.6 years). Respondents most commonly lived in large cities (>500,000 residents, 90/148, 60.8%; 200,000 - 500,000 residents, 27/148, 18.2%; 100,000 - 200,000 residents, 9/148, 6.1%), 12/148 (8.1%) lived in cities up to 50,000 residents, and 10/148 (6.8%) lived in the countryside. Nearly half of the patients had higher education (74/148; 50.0%), 63/148 (42.6%) had secondary education, 6/148 (4.1%) had primary education, and 5/148 (3.4%) of study participants had vocational education. Among the men, 70/148 (47.3%) were married, 53/148 (35.8%) were single, and 25/148 (16.9%) were in a partnership. Of the patients, 125/148 (84.5%) reported having a current sexual partner. The demographic characteristics of the cohort are shown in Table 1."
		(b) Indicate number of participants with missing data for each variable of interest	**5**	"paired BDI-II data were available for 77 participants."
		(c) *Cohort study*—Summarise follow-up time (e.g., average and total amount)	**4**	"Participants were routinely followed up postoperatively, and follow-up questionnaires were

				completed six months after surgery.”
Outcome data	15*	*Cohort study*—Report numbers of outcome events or summary measures over time	**9-11**	“After analyzing the results regarding SXD, a significant increase was observed. Before the procedure, only 10 (6.8%) patients achieved the maximum score for these items. Following this procedure, as many as 20 (13.5%) patients achieved this outcome. All IIEF-15 domains (EF, OF, SXD, IS, and OS) showed clear postoperative improvement in paired analyses (Table 4).”<br><br>“The mean total MGSIS-7 score increased from 19.9 ± 4.5 (median 21; IQR 17–22) preoperatively to 23.1 ± 4.0 (median 21; IQR 21–28) postoperatively (p < 0.001; Table 5).”<br><br>“Postoperatively, the mean total BDI-II score decreased from 1.2 ± 3.8 (median 0) to 0.3 ± 1.6 (median 0) (p = 0.004). Given the very low baseline symptom burden, the high proportion of missing paired BDI-II data, and median scores of zero at both time points, this statistically significant change should be

				considered exploratory and not clinically meaningful.”
		Case–control study—Report numbers in each exposure category, or summary measures of exposure	**-**	n/a
		Cross-sectional study—Report numbers of outcome events or summary measures	**-**	n/a
Main results	16	(*a*) Give unadjusted estimates and, if applicable, confounder-adjusted estimates and their precision (e.g., 95% confidence interval). Make clear which confounders were adjusted for and why they were included	**10,11,15**	“Table 4. IIEF-15 - preoperative and postoperative results.”<br><br>“Table 5. MGSIS-7 - preoperative and postoperative results.”<br><br>“Table S3: BDI-II - preoperative and postoperative results.”
		(*b*) Report category boundaries when continuous variables were categorized	**9**	“The post-treatment classification of patients by ED severity was as follows: 21 (14.2%) were categorized as severe; 2 (1.4%) as moderate; 4 (2.7%) as mild-to-moderate; 23 (15.5%) as mild; and 98 (66.2%) exhibited no ED. Consistently, the mean total IIEF-15 score increased from 46.4 ± 20.4 (median, 53; IQR, 28–63) preoperatively to 57.5 ± 21.0 (median, 68; IQR, 55–70) postoperatively (p < 0.001;”
		(*c*) If relevant, consider translating estimates of relative risk into absolute risk for a meaningful time period	**-**	n/a

Continued on next page

Other analyses	17	Report other analyses done—e.g., analyses of subgroups and interactions, and sensitivity analyses	**-**	n/a
**Discussion**				
Key results	18	Summarise key results with reference to study objectives	**12**	“Our objectives were to characterize patient motivations for surgery, describe perioperative experience […]and evaluate changes in sexual function and selected psychosocial outcomes from the preoperative baseline to postoperative follow-up using a researcher-designed questionnaire and validated instruments […]. Overall, circumcision performed for phimosis was associated with improved patient-reported sexual function […] improved genital self-image, and a modest reduction in depressive symptom scores, accompanied by high satisfaction and a low incidence of clinically significant postoperative complications.”
Limitations	19	Discuss limitations of the study, taking into account sources of potential bias or imprecision. Discuss both direction and magnitude of any potential bias	**13-14**	“however, the very low baseline prevalence of depressive symptoms suggests a floor effect and limits conclusions regarding clinical relevance.”<br><br>“Key limitations include the single-center, observational pre–post design without a control group and

the reliance on self-reported questionnaire data, which are inherently subjective and vulnerable to reporting and response biases. While the observed improvements are statistically robust, the absence of a control group prevents a definitive causal claim; however, the six-month follow-up reduces the likelihood that the findings merely reflect immediate postoperative fluctuations, and it does not eliminate regression to the mean or expectancy effects, which remain important limitations of the uncontrolled pre–post design. Furthermore, the findings may be influenced by selection bias, as the enrollment of a treatment-seeking population might overrepresent individuals predisposed to reporting favorable outcomes. The single-center setting and distinct demographic profile of the cohort also limit the broader external validity of these results. Data completeness was also limited by partial non-response and missing paired follow-up data (particularly for BDI-II), which may reduce generalizability to non-responders despite statistically significant

				findings. In addition, sexual inactivity in a subset of participants represents an important interpretive context for patient-reported sexual outcomes, especially those domains more directly dependent on intercourse.”
Interpretation	20	Give a cautious overall interpretation of results considering objectives, limitations, multiplicity of analyses, results from similar studies, and other relevant evidence	**14**	“Finally, the absence of objective measures of erectile function and biological correlates (e.g., hormonal assessments) limits causal inference; therefore, future research should prioritize multicenter designs with an appropriate control group, objective functional assessments, hormonal analyses, and partner-reported outcomes to further strengthen the evidence base.”
Generalisability	21	Discuss the generalisability (external validity) of the study results	**14**	“Among the strengths of this study are its prospective design and the relatively large cohort (n = 148), which, to the best of our knowledge, represents the largest analysis of this topic in the Polish population and one of the larger European cohorts, thereby addressing an important gap in the literature.”<br><br>“Data completeness was also limited by partial non-response and

				missing paired follow-up data (particularly for BDI-II), which may reduce generalizability to non-responders despite statistically significant findings.”
**Other information**				
Funding	22	Give the source of funding and the role of the funders for the present study and, if applicable, for the original study on which the present article is based	**15**	“Funding: This study received no external funding.”

*Give information separately for cases and controls in case–control studies and, if applicable, for exposed and unexposed groups in cohort and cross-sectional studies.

Abbreviation: n/a, not applicable.

Note: An Explanation and Elaboration article discusses each checklist item and gives methodological background and published examples of transparent reporting. The STROBE checklist is best used in conjunction with this article (freely available on the Web sites of PLoS Medicine at http://www.plosmedicine.org/, Annals of Internal Medicine at http://www.annals.org/, and Epidemiology at http://www.epidem.com/). Information on the STROBE Initiative is available at www.strobe-statement.org.
