# Supplementary material for: Adult Circumcision for Symptomatic Phimosis in Poland: Six-Month Patient-Reported Sexual Function and Psychosocial Outcomes from a Central European Low-Circumcision Setting
Source: J Clin Med. 2026 May 2;15(9):3499. doi: 10.3390/jcm15093499 (PMC13163725; doi:10.3390/jcm15093499)
Supplement: Supplementary file 1 [file jcm-15-03499-s001.zip › Table S3-edited.pdf]

Table S3. BDI-II—preoperative and postoperative results.

	Preoperative (mean ± SD)	Postoperative (mean ± SD)	*p*-value*
1	0.2 ± 0.4	0 ± 0.2	0.006
2	0.1 ± 0.3	0 ± 0.2	0.679
3	0.1 ± 0.4	0 ± 0	0.180
4	0 ± 0.2	0 ± 0	0.109
5	0.1 ± 0.3	0 ± 0.1	0.109
6	0 ± 0.3	0 ± 0	0.180
7	0 ± 0.2	0 ± 0.1	0.361
8	0 ± 0	0 ± 0	not determined
9	0 ± 0	0 ± 0	not determined
10	0 ± 0	0 ± 0	not determined
11	0.1 ± 0.3	0 ± 0.2	0.059
12	0.1 ± 0.3	0 ± 0.2	0.116
13	0.1 ± 0.3	0 ± 0.2	0.423
14	0.1 ± 0.3	0 ± 0.1	0.109
15	0 ± 0.2	0 ± 0	0.180
16	0.1 ± 0.2	0 ± 0.1	0.109
17	0.1 ± 0.4	0 ± 0.1	0.109
18	0 ± 0.3	0 ± 0	0.180
19	0 ± 0	0 ± 0	not determined
20	0 ± 0	0 ± 0	not determined
21	0.1 ± 0.5	0 ± 0.3	0.075
Total	1.2 ± 3.8	0.3 ± 1.6	0.004

* not determined (test not computable due to zero variability in paired responses).
